# Primary Biliary Cirrhosis Associated with Systemic Sclerosis: Diagnostic and Clinical Challenges

**DOI:** 10.1155/2011/976427

**Published:** 2011-12-06

**Authors:** Cristina Rigamonti, Dimitrios P. Bogdanos, Maria G. Mytilinaiou, Daniel S. Smyk, Eirini I. Rigopoulou, Andrew K. Burroughs

**Affiliations:** ^1^Department of Clinical and Experimental Medicine, Università del Piemonte Orientale “A. Avogadro”, 28100 Novara, Italy; ^2^Institute of Liver Studies, King's College London School of Medicine at King's College Hospital, Denmark Hill Campus, London SE5 9RS, UK; ^3^Department of Medicine, University of Thessaly Medical School, Mezourlo, 41110 Larissa, Greece; ^4^The Sheila Sherlock Liver Centre and University Department of Surgery, Royal Free Hospital, London NW3 2QG, UK

## Abstract

Patients with primary biliary cirrhosis (PBC) often have concurrent limited systemic sclerosis (SSc). Conversely, up to one-fourth of SSc patients are positive for PBC-specific antimitochondrial antibodies (AMA). The mechanisms responsible for the co-occurrence of these diseases are largely unknown. Genetic, epigenetic, environmental, and infectious factors appear to be important for the pathogenesis of the disease, but the hierarchy of events are not well defined. Patients with SSc and PBC have an increased morbidity and mortality compared with the general population, but whether the presence of both diseases in an affected individual worsens the prognosis and/or outcome of either disease is not clear. Some case reports suggested that the presence of SSc in PBC patents is associated with a more favorable prognosis of the liver disease, whereas others report an increased mortality in patients with PBC and SSc compared to patients with PBC alone. This paper discusses the features of patients with PBC-associated SSc. Our aims are to clarify some of the pathogenetic, diagnostic, and clinical challenges that are currently faced in the routine management of these patients. We also intend to provide some practical hints for practitioners that will assist in the early identification of patients with PBC-associated SSc.

## 1. Introduction

### 1.1. Primary Biliary Cirrhosis

Primary biliary cirrhosis (PBC) is a chronic cholestatic liver disease characterized by immune-mediated chronic nonsuppurative cholangitis that mainly affects interlobular and septal bile ducts [1–3]. PBC is a rare disease with prevalence ranging from 28 to 402 per million [4], which is highly variable based on geographical location. PBC primarily affects middle aged women [5]. Several reports indicate that the incidence and prevalence of PBC is increasing in the UK, USA, Finland, and Australia [4–7]. PBC often occurs in association with other autoimmune conditions [1–3]. The serological hallmark of PBC is the presence of high-titre serum antimitochondrial autoantibodies (AMA), usually existing in 90–95% of patients with PBC [1–3, 8–16]. The presence of AMA in asymptomatic patients is usually indicative of eventual PBC development [17]. These autoantibodies specifically recognize lipoylated domains within components of the 2-oxoacid dehydrogenase family of enzymes, particularly the E2 component of the pyruvate dehydrogenase complex, located within the inner mitochondrial membrane [1–3, 8–12]. Indirect immunofluorescence using rodent liver, kidney, and stomach sections as substrate is still the most widely used screening assay for AMA in the routine setting [18]. Other techniques such as immunoblotting and ELISA have a higher sensitivity, and the use of cloned mitochondrial antigens and bead assay testing systems allows for the identification of AMA in the sera of patients previously defined as AMA negative [19]. Additionally, PBC-specific antinuclear autoantibodies (ANAs) can be observed in 30% of patients presenting with multiple nuclear dot (antibodies against Sp100) or nuclear membrane staining patterns (antibodies against gp210) [9, 10, 12, 14, 20], which are preferentially identified using HEp-2 cells as substrate [21]. The autoimmune nature of PBC is supported by a plethora of experimental and clinical data, such as the presence of autoreactive T cells and serum autoantibodies in patients with PBC [8, 15, 22–31].

The aetiology of PBC remains unknown, although evidence suggests a role for both genetic susceptibility and environmental factors that remain to be characterized. In fact, a number of chemicals and infectious agents have been proposed to induce the disease in predisposed individuals [22–27, 30, 32–38]. At presentation, patients with PBC may have nonspecific symptoms such as pruritus and fatigue while jaundice is less frequently seen [1–3]. Portal hypertension and its complications may also develop in patients with early, pre-cirrhotic PBC [1–3]. However, the majority of PBC patients are asymptomatic and diagnosed incidentally during the diagnostic workup or treatment for other conditions [39, 40]. Currently, a definite diagnosis of PBC is made on a combination of abnormal serum enzymes indicating cholestasis (i.e., elevated alkaline phosphatase for at least six months), the presence of serum AMA (titre > 1 : 40 by indirect immunofluorescence), and characteristic liver histology with florid bile duct lesions [1–3, 18]. A probable diagnosis is made when two out of these three criteria are present. Serum AMAs (or disease-specific antinuclear antibodies) may precede disease onset by several years, and many individuals found positive for these autoantibodies in the absence of other criteria eventually develop PBC [17].

PBC has a progressive course which may extend over many decades, with greatly variable progression rates among patients. The end of this progression is characterised by cirrhosis, liver failure. However, the patterns of clinical disease and natural history have changed significantly in the last two decades after the introduction of medical treatment with ursodeoxycholic acid (UDCA). When UDCA is administered in early PBC at adequate doses (13–15 mg/kg/day), the progression of the disease is altered, with many patients having a normal life expectancy without additional therapeutic measures [41].

Concomitant autoimmune diseases are often found in patients with PBC. PBC is found in patients with systemic sclerosis (SSc). Also, SSc is one of the most frequent autoimmune rheumatological conditions associated with PBC. This paper discusses the major characteristics of patients with PBC and SSc, and provides clues related to their immunopathogenic link (Table 1).

## 2. PBC-SSc

### 2.1. Epidemiology

PBC has been considered as the most common liver disorder in patients with systemic sclerosis (SSc) [42]. This association was first described to co-occur by Milbradt in 1934, and it has been noted historically in several case reports. One such case from 1964 reports two patients with SSc and possible (but unconfirmed) PBC [43]. Murray-Lyon et al. report two cases of SSc and PBC [44]. The first case was that of a 64-year-old female with Raynauds and scleroderma of the right hand and arm, who was found to have hepatosplenomegaly [44]. She was positive for AMA, and a liver biopsy confirmed the diagnosis of PBC [44]. The second case was similar, with AMA positivity and PBC confirmed on liver biopsy [44]. Despite several similar reports over the years, liver disease has not been considered a significant feature of scleroderma, and a higher prevalence of liver disease was found in the control populations of several large studies [45, 46]. The association of lcSSc and PBC was first described in 1970 with two cases of PBC and limited scleroderma [44]. A further six cases were reported by Reynolds et al. [47], and several other case reports have found an association between lcSSc and PBC. The first case reporting an association of PBC and scleroderma, without features of lcSSc, was described in 1972 [48]. The prevalence of clinically evident PBC among patients with SSc was recently reported to be 2.5% in a registry of 1700 SSc patients [49] and 2% in a series of 817 patients with SSc [50]. On the other hand, the prevalence of SSc in patients with PBC is estimated to be around 8%, as demonstrated by two studies comprising large cohorts of patients with PBC [49, 51]. However, case reports [44, 47, 48, 52–63] and some series reported a wider range of prevalence (3–50%) of SSc, mostly lcSSc, in PBC patients [42, 49, 51, 55, 61, 64, 65].

Large epidemiological studies on PBC note a minority of patients who also have SSc (scleroderma). A large French study found scleroderma in 1% of a cohort of PBC patients, although 1% of their first-degree relatives and 1% of controls were also noted to have scleroderma [66]. One of the most comprehensive epidemiological studies by Gershwin and colleagues found that 2% of PBC patients and 1% of their first-degree relatives had scleroderma, compared to none of the controls [39]. First-degree relatives with scleroderma were more often sisters, followed by daughters of PBC patients [39, 67]. Twin studies in both conditions are scarce. One twin study for SSc found a low concordance of 4.2% among monozygotic (MZ) twins, compared to 5.6% in dizygotic (DZ) twins [68]. Interestingly, there was a 90% concordance for ANA among MZ twins, compared to only 40% among DZ [68]. A higher concordance of 63% among MZ twins was found in the only comprehensive twin study in PBC [69]. Although both twin studies note co-existing autoimmune disease, which was often the same condition in the twin, none have noted SSc in twins with PBC or PBC in twins with SSc.

### 2.2. Immunopathogenesis

Despite the scarcity of case reports and large-scale studies, the association of PBC and scleroderma seems to be more than coincidental and suggests that these two diseases might have a common autoimmune basis. However, the autoimmune mechanisms behind the PBC-SSc association are still not fully understood. It has been reported that this patient group has clonally expanded CD8(+) T cells expressing one T-cell receptor beta-chain variable region, TCRBV3, which may be involved in the disease pathogenesis [70]. Genetic, epigenetic, environmental, and infectious factors appear to be important for the induction of the underlying autoimmune pathology, but the hierarchy of events and the close interplay of these factors are not well defined.

The association between PBC and SSc has been largely based on reports indicating the presence of autoantibodies related to SSc in patients with PBC and *vice versa*. Autoantibodies which characterize limited cutaneous SSc (lcSSC) include anti-centromere antibodies (ACA), anti-Th/To, anti-U1-RNP, and PM/Scl. Diffuse cutaneous SSc (dcSSc) is characterized by anti-Scl 70 antibody (anti-topoisomerase I antibody, TOPO), anti-RNA polymerase III, and anti-U3-RNP [71]. Severe lung disease is the hallmark of anti-TOPO-positive dcSSC patients. DcSSc patients with anti-RNA polymerase III have the most severe skin disease and the highest frequency of renal crisis. Patients with the nucleolar antibody anti-U3-RNP have dcSSc with multiorgan involvement [71].

The autoimmune basis of association between PBC and SSc was first established by the presence of AMA in approximately 5% of patients with scleroderma and ACA in one-quarter of patients with PBC [55]. A positive ACA is reported in 9–30% of PBC patients [59, 72–75] and in 22–25% of all SSc patients, almost all of which have lcSSc. Conversely, up to 25% of SSc patients are AMA positive, but the high prevalence rates of AMA are probably secondary to referral bias and overestimate the frequency of AMA in SSc [76–79]. Another interesting point which needs attention is that of studies reporting a relatively high prevalence of AMA negative PBC in patients with SSc or other autoimmune diseases [51, 80] the autoantibody profile of SSc patients with AMA-negative PBC may require the use of highly sensitive immunoassays for the detection of AMA. It has been shown that such assays are able to detect AMA in serum samples from SSc patients characterized as AMA negative by indirect immunofluorescence, and this may be the case for other PBC-specific autoantibodies, such as ANA specific for sp100 [11, 12, 50].

ACA positivity is greater in PBC-SSc than in either disease in isolation, but there is no cross-reactivity between mitochondrial and centromere antigens [81]. Because ACA have been detected not only in SSc but also in other autoimmune diseases [82–85] including PBC [72, 86], the clinical significance of ACA in PBC has been the focus of ongoing research. Three major centromere antigens have been recognized: centromere protein A (CENP-A, 18 kD polypeptide), centromere protein B (CENP-B, 80 kD polypeptide), and centromere protein C (CENP-C, 140 kD polypeptide). One study attempted to identify the major epitope of ACA in sera obtained from patients with PBC and to classify the correlation between the presence of ACA epitopes and the clinical features in patients with PBC [87]. The serological results obtained were compared with clinical features of lcSSc in PBC. Forty-one patients with PBC were studied: 10 out of 16 (63%) patients with ACA (all anti-CENP A) had one or more lcSSc feature. The higher incidence of Raynaud's phenomenon seen in ACA-positive patients with PBC than that in ACA-negative patients with PBC suggested a close association of the presence of ACA with clinical features of lcSSc in patients with PBC [87]. From the results of this study, it was proposed that there is a subset of PBC patients with scleroderma who are ACA positive and differ from both ACA-negative PBC-SSc and ACA-negative PBC non-SSc patients, based on their clinical features and ACA epitope reactivity [87].

Over the past two years, a tremendous amount of data has come available as to the genetics underlying PBC and SSc. In regards to SSc, several HLA and non-HLA regions have been identified [88], with HLA regions showing variability among ethnic groups. Positive HLA associations in whites and Hispanics include HLA-DRB1*1104, DQA1*0501, DQB1*0301 [89]. Negative associations in those groups included DRB1*0701, DQA1*0201, DQB1*0202, and DRB1*1501 [89]. Positive HLA associations in African Americans included HLA-DRB1*0804, DQA1*0501, and DQB1*0301 [89]. That study also noted that ACA positivity was closely associated with HLA-DQB1*0501 [89], and another study found an association between TOPO positivity and HLA-DRB1*1104 [90]. A smaller study of a Spanish cohort showed similar HLA findings to those noted above [90]. Several non-HLA regions have also been identified in SSc. These include STAT4 [88, 91–94], IRF5 [88, 95, 96], BANK1 [97, 98], TNSF4 [99], TBX21 [92], IL-23R [100], and C8orf13-BLK [101] among others [88]. As with SSc, several HLA and non-HLA regions have been identified in PBC. HLA regions include DRB1, DQA1, DQB1, and DQA2 [102, 103]. Non-HLA regions include IRF5, STAT4, SPIB, IKZF3-ORMDL3, IL12A, IL12RB, MMEL1, DENND1B, CD80, IL7, CXCR5, TNFRSF1A, CLEC16A, and NKFB1 [104–106]. Interestingly, PBC and SSc have several genes in common: HLA-DRB1, DQA1, DQB1, IRF5, and STAT4, although it should be noted that DR11, which is positively associated with SSc, is considered protective in PBC [88, 105].

Infectious agents have been implicated in the pathogenesis of both SSc and PBC. A number of organisms, such as *E. coli*, have been strongly associated with PBC [22, 107, 108], but not with SSc. *Helicobacter pylori* and *Chlamydia* have been implicated in both conditions [109–119]; however, some studies indicate the *Chlamydia* is not involved [72, 120, 121]. It is possible that certain infectious organisms contributes to the development of PBC or SSc in isolation and that other organisms induce the disease in both conditions.

### 2.3. Screening and Diagnosis of PBC in SSc Patients and *Vice Versa*

Given the overlap between PBC with SSc and *vice versa*, including ACA positivity in PBC patients and AMA positivity in SSc patients, the major challenge remains to clarify which screening method would be best for early diagnosis of the associated conditions.

Firstly, routine screening for PBC-specific antibodies in patients with SSc needs to be further refined. Recently, Norman et al. investigated the presence of antibodies against PBC disease-specific mitochondrial antigens and antibodies against the sp100 nuclear body antigen in 52 patients with SSc, by using two commercially available ELISAs [79]. In that study, 13% of cases were positive for AMA and 2% for ANA (anti-sp100), and one patient (2%) was diagnosed with symptomatic PBC [79]. These figures were reproduced by Mytilinaiou et al., who confirmed 13.5% positive results with ELISA testing for antibodies against PBC disease-specific mitochondrial antigens in 37 SSc patients [78]. However, this was not confirmed with the conventional indirect immunofluorescence based on unfixed rodent kidney, liver, stomach tissue sections, or HEp-2 cells as antigenic substrates, and none of the ELISA-positive patients showed features of PBC [78]. It remains to be clarified whether ELISA testing is less specific with false positive results or that it simply represents a more sensitive method with respect to indirect immunofluorescence, which should currently remain the technique of choice.

**Figure 1:**
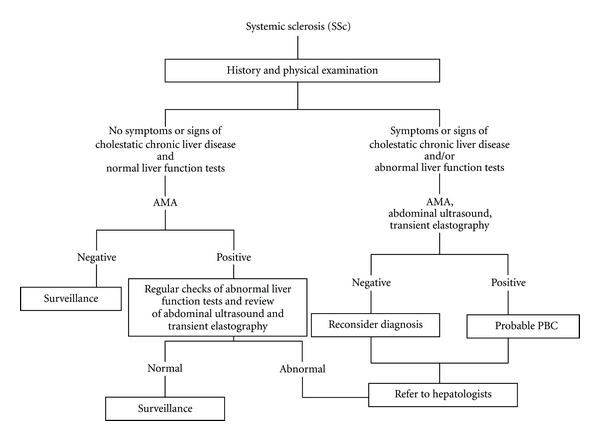
A proposed algorithm for the screening and diagnosis of primary biliary cirrhosis (PBC) in patients with established systemic sclerosis (SSc).

Nevertheless, the presence of AMA can precede clinical symptoms of PBC. Indeed, Mitchison et al. and Metcalf et al. showed that the vast majority of AMA-positive subjects have typical histological features of PBC despite being asymptomatic with normal biochemistry [17, 122]. Furthermore, the study by Prince et al. suggested that 36% of initially asymptomatic PBC patients would become symptomatic within a median time of 5 years [123]. Thus, AMA-positive SSc cases require immediate attention and close, long-term monitoring for early detection of symptoms, signs, and liver biochemistry suggestive of chronic cholestatic liver disease. Routine followup of AMA-positive SSc patients should include liver tests (alanine aminotransferase, aspartate aminotransferase, *γ*-glutamyl transpeptidase, alkaline phosphatase, albumin, bilirubin, international normalized ratio), thyroid function, and possibly an annual ultrasound abdominal scan. Transient elastography of the liver has been used to assess biliary fibrosis in patients with PBC [124]. This test is emerging as a useful screening tool to detect undiagnosed chronic liver disease in apparently healthy subjects [125]. Whether patients with SSc, who are tested positive for PBC-specific AMA, need regular checks with transient elastography or more common tests, such as liver ultrasound, needs to be evaluated in large prospective multicentre studies. Currently, there is no evidence that either of these would be of value.  Figure 1 illustrates the diagnostic and screening algorithm for PBC in SSc patients.

**Figure 2:**
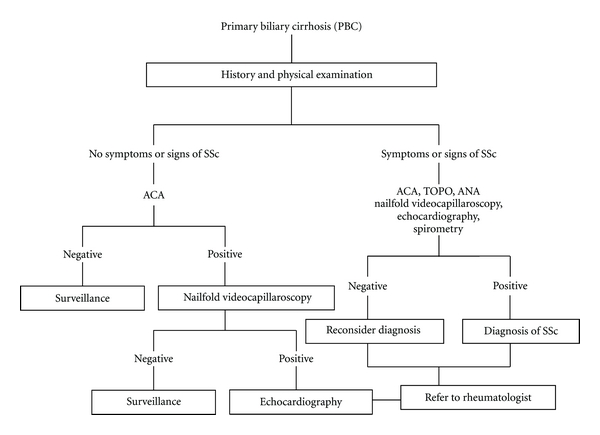
A proposed algorithm for the screening and diagnosis of systemic sclerosis (SSc) in patients with established primary biliary cirrhosis (PBC).

 Screening PBC patients for ACA is not mandatory but can be considered, especially in the presence of disease-related symptomatology. Nakamura et al. reported that, in PBC patients, ACA positivity was significantly associated with more severe ductular pathology on liver histology and was a significant risk factor for the development of portal hypertension [126]. In another study, ACA-positive PBC patients without clinical features of SSc were shown to have similar symptoms and signs at diagnosis [49]. Although ACA positivity is not pathognomic of SSc, it is associated with an increased risk of developing connective tissue disease [127]. One review [128] reported a sensitivity of 32% (17–56%) for SSc and 57% (32–96%) for lcSSc and specificity of at least 93%, while ACA positivity was present in 5% of patients with other connective tissue diseases and less than 1% of disease-free controls. Since ACA could be predictive of rheumatic disorders, it has been suggested that an assessment of PBC patients should always include screening for SSc-related symptoms, such as Raynaud's phenomenon and CREST-related symptoms (calcinosis, Raynaud's phenomenon, esophageal dysmotility, sclerodactyly, and telangiectasia) [129]. The use of nailfold videocapillaroscopy in patients suspected of having connective tissue disease may be a useful indicator. Some evidence suggests that this assessment can be useful for the diagnostic and/or clinical management of patients with PBC and suspected SSc. Experimental and clinical observation suggests that patients with PBC have endothelial dysfunction [130]. In an interesting study, nailfold videocapillaroscopy abnormalities were found in 91% of patients with PBC, and capillary alterations characteristic of SSc were found in 54% [131]. Eleven out of the 22 PBC patients (50%) had extrahepatic signs of connective tissue disease with most being related to SSc, while patients with other types of chronic liver disease did not present with rheumatic manifestations [131]. In PBC patients, there was a significant association between SSc capillary pattern and rheumatic manifestations. The high prevalence of nailfold capillary abnormalities characteristic of SSc in patients with PBC, and correlation with sclerodermal manifestations, suggests that this capillaroscopic finding could be a useful indicator to investigate rheumatic manifestations in these patients [131]. Further clinical assessment of organ involvement (especially lung by spirometry) in association with evaluation of pulmonary artery pressure on echocardiography should be considered in PBC patients with a definite diagnosis of SSc. A proposed diagnostic and screening algorithm for SSc in PBC patients is presented in Figure 2.

### 2.4. Clinical Presentation and Prognosis

In approximately 60% of the cases, the clinical presentation of SSc precedes that of PBC. The demographics of the disease in patients with overlapping features are not well defined. For example, it is not clear whether in the PBC-SSc group the diagnosis of PBC occurs at a lower age than that in patients with PBC alone. In a study of 43 PBC-SSc patients, the median age at diagnosis of PBC made after SSc diagnosis was lower (46.1 years) than in PBC diagnosed before SSc (51.1 years). This was lower than the diagnosis in PBC alone, with a median age of 53.2 years at diagnosis [49]. The different age at diagnosis in the PBC-SSc patients, compared to patients with PBC alone, was probably due to the effect of lead time bias (i.e., screening for PBC in SSc patients and thus early diagnosis of asymptomatic PBC, since 56% presented with SSc alone).

PBC-SSc patients were reported to have a higher incidence of a first episode of spontaneous bacterial peritonitis and septicaemia during followup with respect to patients with PBC alone. This is likely due to an increased risk of infection due to immune abnormalities and organ system manifestations associated with SSc [132].

Both SSc and PBC are associated with increased morbidity and mortality compared with the general population [123, 133–139]. Among the disease-related causes of mortality in SSc patients, pulmonary fibrosis, pulmonary arterial hypertension, and cardiac causes (mainly heart failure and arrhythmias) are reported to account for the majority of deaths. The most frequent non-SSc-related causes of death are infections, malignancies, and cardiovascular causes [140]. In PBC patients, liver-related causes account for roughly 50% of deaths, whereas cardio- and cerebrovascular causes together with malignancies are responsible for the non-liver-related deaths [139, 141]. Some case reports [62, 142] suggest that PBC in association with SSc is associated with a more favourable prognosis than PBC alone, whereas others reported increased mortality due to SSc [143]. In the study which included 43 PBC-SSc patients, liver disease had a slower progression in PBC-SSc compared to matched patients with PBC alone. A lower rate of liver transplantation and liver-related deaths was demonstrated in PBC-SSc patients compared to patients with PBC alone, and these differences were not due to earlier SSc-related deaths [49]. However, the improvement in liver-related survival in the PBC-SSc cohort was outweighed by an increase in non-liver-related deaths due to SSc, and, thus, overall survival was not different in PBC-SSc patients and those with PBC alone [49]. These data emphasize the importance of comorbidity in PBC. More data on the outcome of patients with PBC and PBC with SSc are needed. If patients with PBC and SSc have a lower rate of liver transplantation and liver-related deaths compared to patients with PBC alone [49], it would be expected that patients with PBC and SSc-related ACA would also have better prognosis than their seronegative counterparts, but this does not appear to be the case [126]. It may be that the outcome of patients with ACA-positive PBC, who do not have SSc-related symptoms, differs from that of ACA-positive SSc and PBC overlap.

Prince and colleagues observed an increase in non-hepatic deaths in asymptomatic PBC, even with a reduced liver-related mortality, in comparison with symptomatic PBC [123]. Since the causes of death in PBC-SSc patients are mainly due to SSc and not to liver disease, these patients may need different prognostic models in order to better predict their liver-related survival. Prognostic models for PBC alone may not be applicable for PBC associated with SSc or for other associated autoimmune diseases to assess the risk of liver-related mortality and the need for liver transplantation. 

### 2.5. Therapy

All PBC patients with abnormal liver biochemistry should be considered for specific therapy. UDCA at the dose of 13–15 mg/kg/day on a long-term basis is currently considered the mainstay of therapy for PBC [18]. In the early stages of PBC, UDCA protects injured cholangiocytes against the toxic effects of bile acids. In later stages of the disease, UDCA stimulates impaired hepatocellular secretion, mainly by posttranscriptional mechanisms [144]. In addition, stimulation of ductular alkaline choleresis and inhibition of bile acid-induced hepatocyte and cholangiocyte apoptosis are included among the beneficial effects of UDCA in PBC [144]. UDCA has been demonstrated to markedly decrease serum bilirubin, alkaline phosphatase, *γ*-glutamyl transpeptidase, cholesterol, and immunoglobulin M levels and to ameliorate histological features in patients with PBC in comparison to placebo treatment [145–149]. However, no significant effects on fatigue or pruritus were observed in these large trials nor were effects on survival [150]. Favorable long-term effects of UDCA are observed in patients with early disease and in those with a good biochemical response, which should be assessed after one year from start of treatment [18]. A good biochemical response after one year of UDCA treatment is currently defined by a serum bilirubin ≤1 mg/dL (17 *μ*mol/L), alkaline phosphatase ≤3x ULN, and aspartate aminotransferase ≤3x ULN, according to the ‘‘Paris criteria” [151]. The “Barcelona criteria” indicates a good response with a 40% decrease or normalization of serum alkaline phosphatase [152].

Whether treatment with UDCA has an effect on the symptoms and the outcome of SSc remains poorly understood. Prospective studies of patients with PBC-associated SSc who are followed-up for many years under UDCA treatment are needed to address this issue.

The treatment of SSc is complex and may include drugs with hepatotoxic potential. For example, the use of endothelin-1 receptor antagonist bosentan, which is the treatment of choice for SSc-related pulmonary artery hypertension, has been associated with increased risk of elevated aminotransferases [153–155]. When PBC is present, the management of SSc patients is more challenging, as this autoimmune liver disease may pose further risk factors or unwanted complications. Whichever therapy is to be implemented, it is recommended that collaboration takes place between specialists responsible for the care of these patients.

## 3. Conclusions

The association of SSc and PBC is a rare but intriguing autoimmune syndrome which challenges the expertise and interests of hepatologists and rheumatologists in terms of early diagnosis and shared management. A major effort should be made for continuing collaborative research in this field aimed at achieving a better understanding of the immunopathogenesis, genetic background, and demographic features of patients at higher risk of developing the associated conditions. These findings may also contribute to the development of specific protocols for preventing development and evolution of the two associated diseases.

## Figures and Tables

**Table 1:** Features demographic, immunological and genetic features of primary biliary cirrhosis (PBC) and systemic sclerosis (SSc).

	PBC	SSc
Prevalence (highly variable geographically)	28–402/million	50–200/million
Incidence (highly variable geographically)	2.3–27/million	0.6–122/million
Male to female ratio	1 : 8	1 : 1.5–12 (highly variable geographically)
Peak frequency age	53 years	45–64 years
Autoantibodies	AMA, ANA	*Limited disease*: ACA, anti-Th/To, anti-U1-RNP
		*Diffuse disease*: TOPO, anti-RNA polymerase III, anti-U3-RNP
Genes (positive associations)	*HLA*: DRB1, DQA1, DQB1, DQA2	*HLA*: HLA-DRB1*1104, DQA1*0501, DQB1*0301, HLA-DRB1*0804, DQA1*0501, DQB1*0301
	*Non-HLA*: IRF5, STAT4, SPIB, IKZF3-ORMDL3, IL12A, IL12RB, MMEL1, DENND1B, CD80, IL7, CXCR5, TNFRSF1A, CLEC16A, NKFB1	*Non-HLA*: STAT4, IRF5, BANK1, TNSF4, TBX21, IL-23R, and C8orf13-BLK

AMA, anti-mitochondrial antibody; ANA, anti-nuclear antibody; ACA, anti-centromere antibody.

## Abbreviations

ACA: Anticentromere antibody
AMA: Antimitochondrial antibody
ANA: Antinucluear antibody
CENP-A: Centromere protein A
CENP-B: Centromere protein B
CENP-C: Centromere protein C
dcSSc: Diffuse cutaneous systemic sclerosis
ELISA: Enzyme-linked immunosorbent assay
lcSSc: Limited cutaneous systemic sclerosis
PBC: Primary biliary cirrhosis
SSc: Systemic sclerosis
Anti-TOPO: Anti-topoisomerase (anti-Scl-70) antibody
UDCA: Ursodeoxycholic acid
ULN: Upper limit of normal.
